# Good Samaritan Hospital Medical Clinic

**Published:** 1878-03

**Authors:** 


GOOD SAMARITAN HOSPITAL MEDICAL CLINICS.—*Case of Chorea.*—This little patient, aged 12, is, as you see, unable to walk into the amphitheatre unsupported. This inability to walk is not due, as you may very clearly see, to weakness, because her body and limbs are constantly agitated by violent muscular contractions. These movements are evidently involuntary. There is a lack of the power of co-ordination of the voluntary muscles, and if you were to listen to the heart you would find that the cardiac muscle is also acting irregularly. Just now the cardiac movements are more violent than half an hour ago, when she was in the ward—for coming into the amphitheatre and having the attention of so many persons drawn to her, increases the violence of the symptoms. That is almost invariably the case with patients troubled with chorea, for I need hardly say that this is the disease under which this child is laboring.

The causes of chorea are various. I have succeeded in stopping the disease by the administration of an anthelmintic, the cause of the chorea in such a case being the irritation of the intestinal canal set up by the worms. A sudden fright, or violent emotional disturbance of any kind may be the beginning. The disease seems to be a functional derangement of the nervous centres. We have learned nothing as yet positively concerning the disease from *post mortem* investigations. There seems to be no constant lesion to which we can ascribe the trouble. Hughlings Jackson's theory that the disease depends on minute emboli in the corpora striata, has never yet been proven by the finding of the emboli. He noticed that many of the cases of chorea had valvular disease of the heart, and it is very easy to see how small masses of fibrin may be detached from a valve without vegetation on it, and being carried into the general circulation, finally lodge in the brain. But the theory is simply a theory, and has never yet been proven to be true. The disease often attacks the ill-fed, and those surrounded by bad hygienic influences, but that is not invariably the case, for the most carefully cared for, and those surrounded by every comfort, may exhibit the disease in its worst form. The irregular, spasmodic movements, which is the characteristic feature of the disease, may disappear during sleep, or in bad cases they may continue, making sleep impossible, and finally wearing the sufferer out by exhaustion. It is not uncommon for the skin of the elbows, knees and other prominent parts to be worn away by the violence of the knocks they receive against pieces of furniture, etc.

The treatment in these cases is routine. We must, in the first place, give a tonic, and for these cases there is nothing better, as a usual thing, than arsenic, although iron often does extremely well. The best way to give arsenic is in Fowler's solution, 5 to 10 drops three times a day. We must also try to quiet the nervous centres, and produce sleep. Trosseau has shown that cases of chorea bear morphia in enormous doses. He even speaks of giving 16 grains during a day to a child; but a practitioner would hardly be justified in beginning, at least with such heroic doses. Large doses of chloral are also well borne, and have a very happy effect. I will prescribe for this child five drops of Fowler's solution three times a day. She is to have also ¼ to ½ grain of morphine, commencing first with the smaller dose, with 15 grains of chloral at night.

The hygienic influences surrounding the patient should be made as good as posssible. Where the patient can afford it, a trip to the sea-shore and ocean bathing has often a good effect. The diet should be nourishing, consisting of eggs, milk, beef, etc. The moral management must also be looked to. The patient should be kept quiet, either in a room by itself, or should be at least guarded from the contact with strangers; for if attention is called to the symptoms and the case troubled by observation, the symptoms always become worse. It has even been said that the only thing necessary for the proper treatment of a case of chorea is absolute rest, and retirement to a secluded room, without the use of any medicine whatever. But while I admit the good effects of quiet and rest, I must be better convinced of their curative value, to give up those remedies which undoubtedly have a good effect on the disease.

*Scrofulous Enlargement of the Glands of the Neck.*—Both these patients present the most common symptom of what is known as scrofula. I did not bring them before you in order to enter fully into the disease or state known by that name, or to point out its possible connection with syphilis (for, as you know, some hold scrofula to be merely a symptom of hereditary lues), but simply to speak of the best way in which these glandular enlargements may be treated. If you notice this patient, you see a tumor quite large in the neighborhood of the angle of the lower jaw. It is quite prominent and very disfiguring, and our patient, a young lady, is naturally desirous of having it removed. By palpitation I find no evidence of pus or other fluid; in other words, there is no fluctuation. If left to itself, however, the gland would soon probably break down, and the pus and broken-down-gland substance have to be evacuated. It is, of course, highly desirable to cause the disappearance of this tumor, if possible, before that stage is reached. In the first place, the hygienic surroundings of the patient should be made as good as possible. Exercise in the open air, such as walking or horseback riding, should be ordered. The diet should be looked to, and pastry and other food of that sort interdicted. Sea-bathing often has a good effect; or, when that is not possible, a lump of rock salt dissolved in the morning bath will answer. A tonic should be taken for quite a length of time. Some form of iron is best. The preparation most usually prescribed is, as you know, the syrup of the iodide of iron; but it seems to me that better results are got from the syrup of the double iodides of iron and manganese. Manganese is, as you know, a good tonic in some cases, and this preparation is the most eligible one.

So much for the general treatment; and now what shall we do locally? Our efforts must be directed, of course, to the absorption of this tumor, and the best method I know of is either the injection into the hypertrophied gland of a few drops of iodine or alcohol every few days, or the rubbing in of some drug which we know by experience has the effect desired. If you decide to use the parenchymatous injection of iodide or alcohol, the hypodermic syringe should be charged with 5 to 10 minims of the remedy to be used, the needle thrust deeply into the gland, and the fluid slowly discharged. The needle is then withdrawn, and the finger pressed for a moment over the seat of puncture, in order that the fluid injected may not escape. The first effect of the injection is to cause an inflammation, but after a few days the size of the tumor will be found sensibly decreased. The injection may be repeated when the effects of the first trial have disappeared. If for any cause you cannot, or do not care to use the injection, much good can be done by the application of the ointment of the red iodide of mercury. A small quantity of the ointment should be rubbed in over the tumor every day in the sunlight or near a fire, so that it can be well absorbed. It will be noticed, especially if the skin is tender, that the application causes more or less redness and irritation. Should that be the case, it is well to stop the inunctions for a day or two until the inflammation subsides.

In this little girl which I now show, the destruction and breaking down of the gland tissues has gone so far that we can hope for nothing by either the parenchymatous injection or the inunction of the red iodide ointment. There is evident fluctuation, and that quite superficial. The general treatment is, of course, the same, the idea being simply to build up the general health; but the local treatment is widely different. The pus must be evacuated, and the only question is as to the best way of doing it. If we leave the case to itself, the abscess will burst, and that at no very distant day, and the result will be a large unsightly scar, which will disfigure the child for life. That must, of course, be avoided if possible. If we make a free incision the result will be the same, though not in such a great degree. The method I would advise you to follow in cases like the one before us, is to withdraw the contents of the abscess by aspiration, and then to inject a drachm or so of tincture of iodine, in order to destroy the so-called pyogenic membrane with which the walls are lined. The iodine also sets up an adhesive inflammation, the walls of the abscess are glued together, and the cavity obliterated. By this method you do not leave that puckered, conspicuous scar which is such a common sight on our streets.

*Case of Intestinal Neoplasm.*—This man, aged 46, comes to us complaining of some very significant symptoms, which I shall endeavor to interpret as they are drawn out. You observe that he is quite thin and has a haggard, anxious look. He tells us that when in health his usual weight is 158 pounds. He now weighs 118. His skin, too, has undergone a change. Its color, you observe, is not normal—is distinctly bronzed. The skin is also dry, and lacks that flexibility which is characteristic of it when healthy. He also complains of a dull, heavy, aching pain in the left hypochondrium, and this pain is increased by pressure. By percussion, we find the note high-pitched over the point of tenderness, showing something solid in that region. The spleen can be distinctly marked out, this proving that the solid material, whatever it is, is not connected with that viscus. His appetite, he tells us, is good, but the ingestion of food is followed in about a half hour by a sense of fullness and uneasiness, which is changed in two or three hours to a more distinct feeling of pain, which radiates from the left hypochondrium to the lumbar region. At the same time he hears a gurgling, and experiences the sensation as if a fluid, or rather semi-fluid, mass was being squeezed through a narrow opening. The stools are dark in color and small in calibre. There are distinct glandular enlargements, the cervical as well as other glands being implicated. What is this growth? for that there is a neoplasm there I think is evident. I strongly suspect that it is malignant, and I base my opinion as to that on the cachexia here present and the glandular enlargement. It is not connected with the spleen; that I feel sure of, for reasons already stated. There is sometimes found a cauliflower-growth at about the junction of the transverse with the descending colon, which growth projects into the lumen of the bowel, and narrows its calibre. The feeling he has of distension about one-half hour after eating is very significant, being due to the formation of gas, distending the alimentary canal and bringing pressure on the sensitive point. The feeling of positive pain he complains of in two or three hours after the ingestion of food is very characteristic, the pain being felt at the time the partly digested aliment is passing the narrowed part of the tube. In addition to that, the feeling as if the food were being pressed through a small orifice, and the gurgling he hears at the same time, are strongly confirmatory of the view I have taken. The small size of the fæces must also be remembered, and the enlargement of the cervical glands which often accompanies this form of disease in the abdomen. The treatment is, of course, simply palliative, as we cannot hope to do away with the neoplasm. The diet should be of a character to form as few fæcas as possible, and morphia must be given, preferably hypodermically, in sufficient quantity to relieve the pain.

